# Is the Whole More Than the Sum of Its Parts? Health Effects of Different Types of Traffic Noise Combined

**DOI:** 10.3390/ijerph16091665

**Published:** 2019-05-13

**Authors:** Andreas Seidler, Janice Hegewald, Anna Lene Seidler, Melanie Schubert, Hajo Zeeb

**Affiliations:** 1 Institute and Policlinic of Occupational and Social Medicine, Faculty of Medicine, Technische Universität Dresden, 01307 Dresden, Germany; janice.hegewald@tu-dresden.de (J.H.); lene.seidler@ctc.usyd.edu.au (A.L.S.); melanie.schubert@tu-dresden.de (M.S.); 2 NHMRC Clinical Trials Centre, University of Sydney, NSW 2050 Sydney, Australia; 3 Department of Prevention and Evaluation, Leibniz-Institute for Prevention Research and Epidemiology – BIPS, 28359 Bremen, Germany; zeeb@bips.uni-bremen.de; 4 Health Sciences Bremen, University of Bremen, 28359 Bremen, Germany

**Keywords:** traffic noise, disease risks, epidemiological risk multiplication, energetic noise addition

## Abstract

Many epidemiological studies find that people exposed to aircraft, road or railway traffic noise are at increased risk of illness, including cardiovascular disease (CVD) and depression. It is unclear how the combined exposure to these different types of traffic noise affects disease risks. This study addresses this question with a large secondary data-based case-control study (“NORAH disease risk study”). The Akaike information criterion (AIC) is used to compare two different models estimating the disease risks of combined traffic noise. In comparison with the conventional energetic addition of noise levels, the multiplication of CVD risks as well as depression risks reveals a considerably better model fit as expressed by much lower AIC values. This is also the case when risk differences between different types of traffic noise are taken into account by applying supplements or reductions to the single traffic noise pressure levels in order to identify the best fitting energetic addition model. As a consequence, the conventionally performed energetic addition of noise levels might considerably underestimate the health risks of combined traffic noise. Based on the NORAH disease risk study, “epidemiological risk multiplication” seems to provide a better estimate of the health risks of combined traffic noise exposures compared to energetic addition. If confirmed in further studies, these results should imply consequences for noise protection measures as well as for traffic planning.

## 1. Introduction

Several studies have found an association between aircraft, road or railway traffic noise and cardiovascular diseases [1,2,3,4,5,6,7] as well as depressive disorders [8,9,10,11,12]. The recently published World Health Organization’s (WHO) Noise Guidelines [7] state high quality evidence for a relationship between road traffic noise and the incidence of ischemic heart diseases. Applying a meta-analytic approach, an overall relative risk of 1.08 (95% CI: 1.01–1.15) per 10 dB increase in road traffic noise levels (L_DEN_) was derived across a range of 40 dB to 80 dB. For aircraft noise, the corresponding risk increase in the meta-analysis was 9% per 10 dB; however, the evidence was rated as very low quality. For railway noise, no studies were available and therefore no relative risk estimates could be calculated. One has to point out that the mentioned assessments of the WHO Noise Guidelines are based on the recently published systematic review and meta-analysis of van Kempen et al. [3], which only included epidemiological studies published up to August 2015. Therefore, the later conducted NORAH (Noise-related Annoyance, Cognition, and Health) study on disease risks (which is one of the largest studies in this area to date) was not included in the mentioned meta-analysis. The NORAH study on disease risks confirmed an increased risk of myocardial infarction [4] and heart failure/hypertensive heart disease [5] not only for exposure to road traffic noise, but also to aircraft and railway traffic noise.

Considering the depression risks of traffic noise, several, but not all studies found a positive association. An increased depression risk was found by [8] (road traffic noise), [9] (aircraft, road and railway traffic noise), [10] (aircraft, road and railway traffic noise), [11] (aircraft noise), and [12] (road traffic noise). Overall, whilst there is considerable evidence for increased depression risks due to aircraft noise; the depression risks appear less pronounced for road traffic noise. The only study examining the relationship between railway noise and depression to date was the NORAH study on disease risks [9], which found a statistically significant risk elevation.

All of the previous studies on the disease risks of traffic noise focus on exposure to only one source of traffic noise at a time. However, many people are not only exposed to one type of traffic noise, but to multiple types of traffic noise. For instance, many residential areas under the flight path are also in proximity to major roads or to railroad lines. Little is known about the health effects of such combined exposure to different types of traffic noise, and about the best model to determine these health risks.

The aim of this study was to compare the conventional energetic noise addition model with the epidemiological risk multiplication model and to determine which of the models is better suited to predict health risks of combined types of traffic noise (i.e., the model with the better fit) by calculating the Akaike information criterion (AIC). Our analyses were based on the NORAH study on disease risks, a case-control study comprising routine data of three large statutory health insurance companies around the Rhine-Main Airport [4,5,6,9].

The energetic noise addition model assumes that if different sources of noise are combined, their summed-up noise pressure level reflects the disease risk. Thus, to determine the total sound pressure level resulting from combined exposure to several types of noise, the individual sound pressure levels are summed up energetically. In principle, this would be directly measurable with a standard noise measuring device. For instance, a combined exposure to 60 dB of railway noise and 60 dB of road traffic noise would lead to a measurable noise pressure level of 63 dB (which is equivalent to a doubling of energy, because decibels are on a logarithmic scale), and this noise pressure level of 63 dB would be used in the noise addition model to predict disease risk. However, it is currently unclear if the health effects of this combined exposure can really be equated with the health effects of a 63 dB exposure to one type of traffic noise.

The risk multiplication model, on the other hand, treats different noise sources as separate risk factors and examines their related disease risks, and then, combines these as predictors in a regression model to estimate their combined risk. Thus, it does not simply assess the combination of noise pressure levels, but instead, the combination of multiple disease risk factors (i.e., noise sources). These risks are multiplied since standard epidemiological regression models are based on multiplicative interaction between different risk factors. For instance, assuming the risk increase for traffic noise starts at 40 dB, multiplying the health risks of two different types of traffic noise of 60 dB each would result in a combined risk equivalent to the health risk of one type of traffic noise alone of 80 dB: 40 dB (the baseline risk) plus two times 20 dB (i.e., the excess risk at 60 dB is calculated as the difference 60 dB – 40 dB).

For the noise addition model, first a combined noise pressure level is calculated and then the risk of this combined noise pressure level is estimated. For the risk multiplication model, first the risk of each traffic noise level is estimated independently and then the combined risk of these separate risk factors is estimated. For both models, a so-called noise equivalent level is calculated that reflects the noise pressure level from one noise source at which the same disease risk would be expected, as found for exposure to multiple noise sources.

Which model is more accurate in determining disease risks of combined exposure has important implications for the resulting disease risks: If disease risks of combined exposure to different sources of traffic noise multiplied (risk multiplication model), these disease risks could be considerably higher than predicted by the conventional energetic addition of noise pressure levels (energetic addition model). This would imply consequences for noise protection measures as well as for traffic planning: Separating roads and railway lines might sometimes better prevent noise-related diseases than the (frequently favored) parallel routing of roads and railway lines.

## 2. Materials and Methods

The study population of the NORAH (Noise-related Annoyance, Cognition, and Health) study on disease risks consisted of about 1 million individuals that were insured by three large statutory health insurance funds in the Rhine-Main area of Germany. The study design has been described in detail elsewhere [4,5,6,9]. Briefly, based on insurance claims and prescription data, 130,945 cases (59,182 males, 71,763 females; median age 73 years) of cardiovascular diseases diagnosed between 2006 and 2010 were identified and compared with control subjects not suffering from cardiovascular diseases (280,809 males, 355,353 females; median age 56 years). To be included in the case group, one or more of the following cardiovascular diseases had to be diagnosed: Myocardial infarction, stroke, heart failure and/or hypertensive heart disease. As a second case group, 77,295 depression cases (24,914 males, 52,381 females; median age 59 years) and appropriate control subjects (292,239 males, 286,007 females; median age 61 years) were analyzed. Address-specific exposure to aircraft, road and railway traffic noise in 2005 was estimated for all cases and control subjects. The study was approved by the Ethics Committee of the Medical Faculty, TU Dresden (EK328102012). 

### 2.1. General Analytic Approach: Calculation of Risk Increase per 10 DB Traffic Noise Applying a Linear Model

The best fitting model would be the model that best captures the structure of the analysed data. Overall noise level equivalents were calculated to combine the exposure to the three different types of traffic noise studied into one exposure variable. In the conventional energetic addition model, to calculate the noise level equivalent, the noise pressure levels of the single traffic noise levels were energetically summed up. In the risk multiplication model, the noise level equivalent was defined as the exposure to a single traffic noise pressure level of which the estimated disease risk equals the disease risk of the product of the single traffic noise-specific disease risk estimates. In contrast to the risk multiplication model, in the energetic addition model, the calculation of the noise level equivalent is based on traffic noise pressure levels, not on traffic noise-specific risks. That means that potential risk differences between different types of traffic noise can only be compensated by applying supplements or reductions to the single traffic noise pressure levels. To ensure that differences in the model fit can really be attributed to the basic properties of the two models under comparison (conventional energetic addition model vs. risk multiplication model) and not to specific risk differences between different types of traffic noise, we therefore first optimised the model fit of the energetic addition model by applying correction values. Subsequently, we compared the model fit applying the noise level equivalents of the energetic addition model and applying the noise level equivalents of the risk multiplication model.

As a general analytic approach, odds ratios (OR) with 95% confidence intervals (CI) were calculated using logistic regression analysis, adjusted for age, sex, local proportion of individuals receiving unemployment benefits, and individual socioeconomic status (if available). For depression, we additionally included urban living environment in the logistic regression analyses.

In accordance with several systematic reviews e.g., [1,2,3], we assumed a linear exposure-response relationship between traffic noise exposure and the diagnosis of the studied diseases, choosing a starting point of 40 dB of 24 h continuous sound levels (L_pAeq,24h_). In fact, for most of the considered diseases, we could show an adequate model fit for a linear model in the NORAH study on disease risks. Only for depression, the exposure-risk relation revealed a reverse “U-curve” for aircraft and railway noise, possibly due to selection effects (“healthy resident effect”, see [9]). However, a healthy resident effect would not reflect real effects of traffic noise and should therefore not be included in the risk assessment. Therefore, the assumption of a linear exposure-risk relation appears justifiable, even with the (few) exceptions highlighted. As the NORAH study on disease risks found comparable risks for men and women as well as for older and younger individuals, we did not stratify for sex or age.

### 2.2. Dealing with Risk Differences between Different Types of Traffic Noise

It is unclear if the disease risks associated with traffic noise differ between different types of traffic noise. To date, few studies have analyzed the disease risks of traffic noise applying the same methodology for different types of traffic noise. The above mentioned WHO review found risks of roughly comparable magnitude for road traffic noise and aircraft noise, but no studies on the risk of rail traffic noise could be identified. Overall, no clear conclusion about differences in health risks across individual types of traffic noise can be derived from this WHO review. We therefore applied two different approaches to combining the noise levels of different types of traffic noise into one overall noise-level variable (“noise level equivalent”).

The first approach assumed equal disease risks for the various types of traffic noise. The second approach took risk differences for different types of traffic noise into account by using the risk differences across types of traffic noise that we found in the NORAH study on disease risks. We introduced correction values (see Section 2.3) to ensure that differences in the model fit can only be attributed to the basic properties of the two models under comparison (energetic addition model vs. risk multiplication model), not to specific disease risk differences between different types of traffic noise. We examined to what extent the results of this second approach are compatible with the results of the first approach by comparing the model fits.

To combine the exposure to three different types of traffic noise exposure into one exposure variable, we calculated overall noise levels. In the following, these are called “noise level equivalents“, as they do not constitute real measurable noise pressure levels. These noise level equivalents are only suitable to reflect the risks of the disease under consideration, not the risks of other diseases as, for example, noise-induced hearing loss.

### 2.3. Calculation of Overall Noise Level Equivalents Based on Energetic Addition of Different Types of Traffic Noise

The noise level equivalent based on energetic addition was calculated using the following formula (first approach assuming equal disease risks):

Noise level equivalent = 10 × log10(10^aircraft noise/10^ + 10^road traffic noise/10^ + 10^railway noise/10^) (1)

where noise levels were set from <40 dB to 40 dB.

Compared with road traffic noise, the NORAH study on disease risks found higher cardiovascular risks for railway noise and lower cardiovascular risks for aircraft noise (Table 1, upper section). To best reflect the noise-type-specific risks, we added different correction values: the correction values were increased in 1 dB steps for railway noise (+1, +2, …, +20) and decreased in 1 dB steps for aircraft noise (−1, −2, …, −20) to find the best fitting energetic addition model. Correction values were only applied for uncorrected noise levels of >40 dB. Correction values were limited to values between −20 and +20 to avoid unrealistic noise levels. If no clear AIC minimum could be achieved within this range of correction values (−20 … +20), as a sensitivity analysis, the correction values were lowered or elevated until an AIC minimum could be found (see Section 3. Results). The model with a correction value of +5 dB for railway noise and −20 dB for aircraft noise revealed the lowest AIC value (see Section 2.4) and was therefore the best fitting energetic addition model for cardiovascular diseases.

In the NORAH study on disease risks, depression risks were highest for aircraft noise and lowest for road traffic noise (Table 2, bottom section). We therefore again determined the best energetic addition model (defined by the model with the lowest AIC value) by increasing the correction value for aircraft noise (+1, +2, …, +20) and decreasing the correction value for road traffic noise (−1, −2, …, −20) in 1 dB steps; this only resulted in a correction value for aircraft noise of +20 dB.

The corrected noise level equivalent based on energetic addition was calculated using the following formula (second approach):

Noise level equivalent = 10 × log10(10^(aircraft noise+a)/10^ + 10^road traffic noise+b/10^ + 10^(railway noise+c)/10^) (2)

where the above mentioned correction values are *a* for aircraft noise, *b* for road traffic noise, and *c* for railway noise, again setting noise levels <40 dB to 40 dB.

### 2.4. Calculation of Overall Noise Level Equivalents Based on Epidemiological Risk Multiplication of Different Types of Traffic Noise

Risk estimates for single types of traffic noise are required to apply the epidemiological risk multiplication model. Based on our analytic approach described in Section 2.1, we therefore initially calculated ORs including the single types of traffic noise exposure in separate regression models. For cardiovascular disease, there was a risk increase of 1.0% per 10 dB aircraft noise, 2.4% per 10 dB road traffic noise, and 3.6% per 10 dB railway noise (Table 1, top section). For depression, the risk increase was 13.0% per 10 dB aircraft noise, 4.1% per 10 dB road traffic noise, and 5.8% per 10 dB railway noise (Table 1, bottom section).

In the first approach, the overall noise level equivalents were calculated based on equal disease risks for all types of traffic noise. The median noise-related cardiovascular risk increase which was a risk increase of 2.4% per 10 dB road traffic noise was attributed to all types of traffic noise. For analyses of depression risk, the railway noise-related risk of 5.8% per 10 dB (which was the median noise-related depression risk of the three types of traffic noise) was attributed to all types of traffic noise. In the second approach, the overall noise level equivalents were calculated based on the above-mentioned noise type-specific risks (Table 1).

The noise level equivalent for cardiovascular diseases based on epidemiological multiplication of equal disease risks was calculated using the following formula (first approach using equal NORAH risk estimates):

Noise level equivalent = 10 × ((log10(1.024aircraft noise/10−4 × 1.024^road traffic noise/10–4^ × 1.024^railway noise/10−4^)/log10(1.024)) + 4), (3)

where noise levels were set to <40 dB to 40 dB.

The noise level equivalent for depression based on epidemiological multiplication of equal risks was calculated using the following formula (first approach using equal NORAH risk estimates):

Noise level equivalent = 10 × ((log10(1.058^aircraft noise/10−4^ × 1.058^road traffic noise/10−4^ × 1.058^railway noise/10−4^)/log10(1.058)) + 4), (4)

where noise levels were set to <40 dB to 40 dB.

The noise level equivalent for cardiovascular diseases based on epidemiological multiplication of noise type-specific risks was calculated using the following formula (second approach assuming traffic-specific disease risks):

Noise level equivalent = 10 × ((log10(1.010^aircraft noise/10–4^ × 1.024^road traffic noise/10−4^ × 1.036r^ailway noise/10−4^)/log10(1.024)) + 4), (5)

where noise levels were set to <40 dB to 40 dB.

The noise level equivalent for depression based on epidemiological multiplication of noise type-specific risks was calculated using the following formula (second approach assuming traffic-specific disease risks):

Noise level equivalent = 10 × ((log10(1.13^aircraft noise/10–4^ × 1.041^road traffic noise/10−4^ × 1.058^railway noise/10−4^)/log10(1.058)) + 4), (6)

where noise levels were set to <40 dB to 40 dB.

### 2.5. Comparison of Regression Models Applying Energetic Addition vs. Risk Multiplication by Akaike Information Criterion (AIC)

With the noise level equivalents as (single) exposure variables, we again analyzed the association between traffic noise exposure and cardiovascular diseases or depression. We calculated ORs as described under Section 2.1, using the stated confounder sets.

We applied the Akaike information citerion (AIC) to compare the adequacy of the energetic addition vs. epidemiological risk multiplication models describing combination effects of aircraft, road and railway traffic noise. The AIC provides a measure of the suitability of a statistical model (the goodness of fit) for a given dataset [13]. The smaller the value of the AIC, the better the model fits the data. The AIC value cannot be interpreted directly. As a rough rule of thumb, the empirical support is “essentially none” that a model with an AIC difference of more than 10 compared with the lowest found AIC value in fact constitutes the “best model” [13] (p. 70). The two competing models, and the difference in the derived disease risk depending on model choice, will be illustrated in more detail at the end of the results section using an example of combined noise exposure and the derived disease risks.

## 3. Results

### 3.1. Cardiovascular Risks of Combined Traffic Noise Exposure

When the sound pressure levels of aircraft, road traffic and railway noise are energetically summed up (Table 2, top section) without any supplements or reductions, the risk of cardiovascular diseases is elevated by 2.9% per 10 dB. This model serves as the “basic model”. In our study population, the highest “noise level equivalent” of 85.7 dB can be found in individuals that are exposed to 85.7 dB road traffic noise, but not to aircraft noise risk or railway noise above 40 dB. The highest risk increase for this basic model—related to a noise level equivalent of 85.7 dB—is calculated as a 14% increase compared to unexposed individuals (aircraft, road and railway noise levels ≤40 dB). The best fitting energetic addition model resulting from a reduction of 20 dB for aircraft noise and an addition of 5 dB for railway noise considerably improves the model fit as expressed by an AIC difference of −10.2. In this model, the highest noise level equivalent of 88.9 dB can be found for a combination of 44.9 dB aircraft noise, 50.5 dB road traffic noise and 83.9 dB railway noise. The combined noise exposure in this example leads to a risk increase of 15% compared to unexposed individuals. If, in a sensitivity analysis, the aircraft noise correction factor was further decreased, this did not lead to lower AIC values.

In comparison to the energetic summation of the noise pressure levels, the epidemiological risk multiplication (Table 2, bottom section) reveals a considerably better model fit as expressed by lower AIC values. Compared with the basic model, the epidemiological risk multiplication assuming equal risk estimates for the three types of traffic noise improves the model fit by −12 AIC points. The highest noise level equivalent of 119.2 dB corresponds to a combined exposure of 61.7 dB aircraft noise, 75.5 dB road traffic noise and 72.0 dB railway noise.

It should be noted that the risk increase per 10 dB cannot be directly compared between the “energy summation model” and the “epidemiological risk multiplication model”, as the resulting risk increase also depends on the noise level equivalents. In other words, based on high noise level equivalents resulting from epidemiological risk multiplication, even relatively small risk increases per 10 dB (compared to the energetic addition model) for high noise level equivalents may lead to large increases in total risk. Thus, in the previously mentioned epidemiological risk multiplication model, the highest calculated total risk increase observed in the study population is 19%, which is higher than the highest risk increase in all energetic addition models. When the observed noise type-specific cardiovascular risk increase is taken into account, the highest risk increase is elevated to 22%; and the model fit further improves, expressed by an AIC difference of −22.1 compared to the basic model and by an AIC difference of −11.9 (−22.1 minus −10.2) compared to the best fitting energetic addition model. The highest noise level equivalent of 134.8 dB corresponds to a combined exposure of 47.0 dB aircraft noise, 71.2 dB road traffic noise and 80.7 dB railway noise. Overall, risk multiplication models provide a considerably better fit of the traffic noise-related cardiovascular risks than energetic addition models.

### 3.2. Depression Risks of Combined Traffic Noise Exposure

The much better fit of the risk multiplication model is even more pronounced for depression risks (Table 3), as indicated by the much lower AIC values when comparing the risk multiplication models to the basic model: the epidemiological risk multiplication assuming equal risk estimates for the three types of traffic noise (Δ AIC = −108.6) strongly improves the model fit (Table 3, bottom section) compared with the basic model. Adding +20 dB for aircraft noise considerably improves the energetic addition model fit as expressed by an AIC difference of –72.8 to the basic model (Table 3, top section). By adding a correction value of up to +20 dB aircraft noise, no AIC minimum could be reached. If, in a sensitivity analysis, the aircraft noise correction factor was further increased up to an (unrealistic) high value of +200 dB, with an AIC difference of –148.2 to the basic model, the best fitting energetic model would fit better than both of the risk multiplication models. Applying a risk-multiplying approach in our study population, we calculated the highest depression risk increase of 47% for a combined exposure to 61.3 dB aircraft noise (24 h sound level), 53.4 dB road traffic noise and 70.7 dB railway noise. The highest depression risk increase for the basic energetic sound-pressure addition model would be at only 27% (for an exposure to 85.7 dB road traffic noise with aircraft noise and railway noise lying below 40 dB).

### 3.3. Example of Risk Increase for CDV for the Two Different Models

Table 4 illustrates the risk increase predicted by the two different models for the example of 50 dB aircraft, 60 dB road traffic and 70 dB railway traffic noise, and with and without correcting for differences between different traffic types. The predicted risk increase varies across the different models—the energetic addition model predicts a lower risk increase for combined exposure to multiple sources of traffic noise than the risk multiplication model. Applying correction factors further increases the predicted risk in this example.

## 4. Discussion

In the NORAH study on disease risks, the conventional energetic addition model does not adequately reflect cardiovascular and depression risks by combined types of traffic noise. The epidemiological risk multiplication model of traffic noise-related disease risks revealed a much better model fit for cardiovascular diseases as well as for depression than the conventional energetic addition model. Thus, we were able to confirm Aristotle’s thesis with respect to the health effects of traffic noise: “… B and A are not the same thing as BA …” [14], simply put: The whole is more than the sum of its parts. As shown in the example (Table 4), the better-fitting risk multiplication model may reveal a considerably higher risk increase than the energetic addition model.

### 4.1. Strengths and Limitations

As a strength of our novel approach, we were able to compare the model fit of two different ways of assessing the risk of combined traffic noise exposure on the basis of a large case-control study. For each of the three different types of traffic (road, railway traffic, and aircraft), continuous noise measures had precisely been assessed for each address in the study area, using most recent international guidelines and multiple information sources.

Since evidence to-date is inconclusive on whether and to what extent disease risks differ between different types of traffic noise (see [3,7]), we assessed a basic model assuming equal risks for different types of traffic noise. By additionally introducing correction values into the energetic addition model, we optimized the model fit of the energetic addition model, allowing for a true comparison of the basic properties of the two analyzed models (conventional energetic addition model vs. risk multiplication model). As expected, we found that including correction factors in the models improved the model fit.

Our analyses were limited by the lack of more detailed temporal data: We do not know to what extent there was real combined exposure to several types of traffic noise at the same daytime or nighttime. Moreover, our analyses were based on 2005 traffic noise data. In fact, disease risks of traffic noise might depend on long-lasting cumulative exposure. If an individual, for example, had moved shortly before 2005 from a noisy to a relatively quiet residential address (or vice versa), the effective exposure might have been underestimated (resp. overestimated) in our analysis.

Furthermore, we were not able to take other types of noise exposure such as occupational noise into account. Moreover, our study population is characterized by relatively high traffic noise exposure. Our study results cannot simply be transposed to lower-exposed regions or to regions with different noise characteristics.

### 4.2. Methodological Considerations

The analysis of depression risks of combined types of traffic noise revealed a best fitting model with an unrealistic correction value of more than +200 dB aircraft noise (see Table 3, second footnote). In fact, when applying this unrealistic high correction value, the energetic addition model would fit even better than the epidemiologic risk multiplication model. A potential explanation for the best fit with extremely high aircraft noise level equivalents lies in the nonlinear exposure-response relationship between aircraft noise and depression. When this nonlinear exposure-response relationship is forced to be represented by a linear model, the steep risk increase at low aircraft noise levels might be best reflected by very high noise level equivalents. As an alternative—or additional—explanation, the aircraft-noise related depression risks at relatively low aircraft noise levels might be so dominating that combined exposure to road or railway noise would not have a substantial effect on the depression risk. This would mean that the dominant source model (see Section 4.3) could potentially contribute to the understanding of the effects of aircraft noise on depression onset. However, the before-mentioned explanations are rather speculative; therefore, the appropriateness of different explanatory models of the combined effects of different types of traffic noise should be investigated in further large-scale studies.

### 4.3. Interpretation and Implications

To our knowledge, this is the first study of its kind to analyze the disease risks of combined types of traffic noise. To date, different models have been examined only for the outcome of annoyance by combined exposure to different types of traffic noise. In annoyance studies, just as in our basic model, the energetic addition model [15] assumes that each of the singular noise sources leads to the same annoyance at equal noise levels. Similar to our model applying correction values, the annoyance equivalents model “translates the noise from the individual sources into the equally annoying sound levels of a reference source, road traffic, and then sums these levels“ [16], giving a total sound pressure level. This total sound pressure level corresponds to our noise level equivalents and substitutes the road traffic noise exposure in the road traffic exposure–annoyance relationship. A different approach is taken in the so-called dominant source model [17]. According to this dominant source model, the most annoying source of traffic noise (sometimes understood as the loudest source) determines the total noise annoyance of combined types of traffic noise. At first glance, our finding of a multiplication of disease risks seems to be in strong contrast to this dominant source model: According to the dominant source model, in case of one clearly dominating source of traffic noise, the other noise sources could rather be neglected when determining total noise annoyance. In this case, the total annoyance could be even (slightly) lower than predicted by the energetic addition model. A potential explanation for these somewhat contrasting findings might lie in at least partly different pathophysiologic mechanisms of annoyance and cardiovascular diseases. Not only could conscious noise effects, such as annoyance, be of importance for the noise-related development of cardiovascular diseases, but unconscious noise effects such as disturbance of sleep may be important.

In our study region, there was a strong association between socioeconomic status and exposure to multiple types of traffic noise: individuals in the poorest areas were ten times more likely to be exposed to high levels of all three types of traffic noise combined than individuals living in the richest areas. Thus, an already vulnerable population, due to its limited socioeconomic resources, might be at considerable further risk of CVD and depression due to combined traffic noise. This is particularly important for environmental justice considerations [18].

## 5. Conclusions

Cardiovascular risks as well as depression risks of combined exposure to different types of traffic noise seem to be considerably higher than what would be expected from the conventional approach of simply adding up individual sound pressure levels. If confirmed, the finding of particularly high disease risks associated with exposure to combined noise from multiple types of traffic has particular importance for prevention. Traffic planning should take into account the risks resulting from combined exposure of the residential population to multiple types of traffic noise. This could mean avoiding parallel routing of roads and railway lines in populated areas. However, it is important to point out that in order to provide a sound basis specifically for the derivation of concrete traffic-noise induced disease risks and preventive measures, further large-scale epidemiological studies will be required, specifically focusing on the effects of combined exposure to different types of traffic noise.

## Figures and Tables

**Table 1:** Risk increase for cardiovascular increases and depression per 10 dB traffic noise (starting point: 40 dB).

Traffic Noise	Exposure	Risk Increase (OR ^1^) per 10 dB	95% CI
Cardiovascular diseases			
Aircraft noise	L_pAeq,24h_	1.0%	−0.8–2.8%
Road traffic noise	L_pAeq,24h_	2.4%	1.6–3.3%
Railway noise	L_pAeq,24h_	3.6%	2.4–4.7%
Depression			
Aircraft noise	L_pAeq,24h_	13.0%	10.7–15.4%
Road traffic noise	L_pAeq,24h_	4.1%	3.1–5.0%
Railway noise	L_pAeq,24h_	5.8%	4.5–7.1%

^1^Adjusted for age, sex, local proportion of individuals receiving unemployment benefits, individual socioeconomic status (if available), urban living environment (for depression).

**Table 2:** Risk increase for cardiovascular diseases per 10 dB noise level equivalent (starting point: 40 dB).

Traffic Noise (L_pAeq,24 h_)	Correction (values)	Highest Noise Level Equivalent	Risk increase (OR ^1^) per 10 dB Noise Level Equivalent (95% CI)	Highest Risk Increase	AIC Difference to the “Basic Model”
Energetic addition of aircraft, road traffic and railway noise					
“Basic model”	None	85.7 dB	2.9% (2.0–3.8%)	14%	-
	Aircraft –20 dB, railway +5 dB	88.9 dB	3.0% (2.2–3.8%)	15%	–10.2
Multiplication of the single traffic–noise related risk estimates					
	2.4% risk increase for all noise types	119.2 dB ^2^	2.1% (1.6–2.7%)	19%	–12.0
	Type-specific ^3^ risk increases	134.8 dB ^2^	2.1% (1.6–2.6%)	22%	–22.1

^1^Adjusted for age, sex, local proportion of individuals receiving unemployment benefits, individual socioeconomic status (if available). ^2^Converted to the “noise effect” of road traffic noise: the highest calculated level equivalent value indicates the road traffic noise level that would be associated with the calculated highest risk increase in the case of sole road traffic noise exposure. Further decrease of the aircraft noise correction factor would not lead to lower AIC values. ^3^For aircraft noise: 1.0% per 10 dB; for road traffic noise: 2.4% per 10 dB, for railway noise: 3.6% per 10 dB.

**Table 3:** Risk increase for depression per 10 dB noise level equivalent (**starting** point: 40 dB).

Traffic Noise (L_pAeq,24 h_)	Correction (Values)	Highest Noise Level Equivalent	Risk Increase (OR ^1^) per 10 dB Noise Level Equivalent (95% CI)	Highest Risk Increase	AIC Difference to the “Basic Model”
Energetic addition of aircraft, road traffic and railway noise					
“Basic model”	No	85.7 dB	4.8% (3.7–5.8%)	24%	–
	Aircraft +20 dB ^2^, road no correction	85.7 dB	5.4% (4.6–6.3%)	27%	–72.8
Multiplication of the single traffic–noise related risk estimates					
	5.8% risk increase for all noise types	119.2 dB	4.7% (4.0–5.3%)	44%	–108.6
	Type–specific ^3^ risk increases	126.3 dB ^3^	4.6% (4.0–5.2%)	47%	–138.3

^1^ Adjusted for age, sex, local proportion of individuals receiving unemployment benefits, individual socioeconomic status (if available), urban living environment. ^2^ Converted to the “noise effect” of railway noise: the highest calculated level equivalent value indicates the railway noise level that would be associated with the calculated highest risk increase in the case of sole railway noise exposure. Further increase of the aircraft noise correction factor up to an unrealistic high value of 200 dB would lead to a further decrease of the AIC to about −148. ^3^ For aircraft noise: 13.0% per 10 dB; for road traffic noise: 4.1% per 10 dB, for railway noise: 5.8% per 10 dB.

**Table 4:** Example of the different disease risks derived by the two different models for the example scenario with 24 h-continuous noise pressure levels for aircraft noise of 50 dB, road traffic noise of 60 dB, and railway noise of 70 dB.

Scenario of Three Sources of Traffic Noise Combined: Aircraft Noise of 50 dB, Road Traffic Noise of 60 dB, and Railway Noise of 70 dB	Model	Correction to Consider Study-Specific CVD Risk Differences between Different Sources of Traffic Noise	One-Source Noise Level Equivalent	Risk Increase (OR) per 10 dB Increase of One-Source Noise Equivalent According to Logistic Regression Analysis	Overall Relative CVD Risk (and Relative CVD Risk Increase) in the Considered Scenario
	**Energetic addition**	None (basic model)	Overall noise level of the assumed three sources combined = 10 × log10(10^50 dB aircraft noise/10^ + 10^60 dB road noise/10^ + 10^70 dB railway noise/10^) = 70.5 dB (*formula 1*)	2.9% (*Table 2*)	Overall relative risk estimate = 1.029^noise level equivalent/10−4^ = 1.029^70.5 dB/10−4^ = 1.091 (**risk increase of 9.1%**)
		+5 dB for railway noise; −20 dB for aircraft noise	Overall (road traffic) noise level of three sources of “corrected” single noise levels = 10 × log10(10^40 dB aircraft noise/10^ + 10^60 dB road noise/10^ + 10^75 dB railway noise/10^) = 75.1 dB (*formula 2*)	3.0% (*Table 2*)	Overall relative risk estimate = 1.030^noise level equivalent/10−4^ = 1.030^75.1 dB/10−4^ = 1.109 (**risk increase of 10.9%**)
	**Risk multiplication**	Equal risk increase of 2.4% per 10 dB increase of each noise level (according to risk increase of road traffic noise, *Table 1*)	One-source noise level that would comprise the same CVD risk as the assumed sources combined = 10 × ((log10(1.024^50 dB aircraft noise/10–4^ × 1.024^60 dB road traffic noise/10–4^ × 1.024^70 dB railway noise/10–4^)/log10(1.024)) + 4) = 100 dB (*formula 3*)	2.1% (*Table 2*)	Overall relative risk estimate = 1.021^noise level equivalent/10−4^ = 1.021^100 dB/10−4^ = 1.133 (**risk increase of 13.3%**)
		Type-specific risk increase of 1.0% per 10 dB increase of aircraft noise, 2.4% per 10 dB increase of road traffic noise, and 3.6% per 10 dB increase of railway noise (*Table 1*)	One-source (road traffic) noise level that would comprise the same CVD risk as three sources of 60 dB each based on study-specific risk findings = 10 × ((log10(1.010^50 dB aircraft noise/10–4^ × 1.024^60 dB road traffic noise/10–4^ × 1.036^70 dB railway noise/10–4^)/log10(1.024)) + 4) = 108.9 dB (*formula 5*)	2.1% (*Table 2*)	Overall relative risk estimate = 1.029^noise level equivalent/10–4^ = 1.021^108.9 dB/10–4^ = 1.154 (**risk increase of 15.4%**)
